# Recent Advances in Synthetic Chemical Inducers of Plant Immunity

**DOI:** 10.3389/fpls.2018.01613

**Published:** 2018-11-06

**Authors:** Mian Zhou, Wei Wang

**Affiliations:** ^1^Department of Plant Pathology and Microbiology, Iowa State University, Ames, IA, United States; ^2^School of Life Sciences, Peking University, Beijing, China; ^3^Peking University – Tsinghua University Joint Center for Life Sciences, Beijing, China

**Keywords:** plant immunity, plant immune inducers, chemical derivation, ionic liquids, diversity-oriented synthesis, computer-aided design

## Abstract

Different from the conventional biocidal agrochemicals, synthetic chemical inducers of plant immunity activate, bolster, or prime plant defense machineries rather than directly acting on the pathogens. Advances in combinatorial synthesis and high-throughput screening methods have led to the discovery of various synthetic plant immune activators as well as priming agents. The availability of their structures and recent progress in the mechanistic understanding of plant immune responses have opened up the possibility of identifying new or more potent chemical inducers through rational design. In this review, we first summarize the chemical inducers identified through large-scale screening and then discuss the emerging trends in the identification and development of novel plant immune inducers including natural elicitor based chemical derivation, bifunctional combination, and computer-aided design.

## Introduction

While plants are important nutritional source of humans, they are also consumed by various heterotrophic microorganisms, which cause diverse plant diseases and considerable economic loss to agriculture. To reduce the yield loss, conventional chemical pesticides have been developed. They exert their functions through direct biocidal effects on the pathogens. However, besides the toxicity on the pathogens, conventional pesticides may also have negative impacts on the crops, beneficial microorganisms and the health of farmers and consumers. Furthermore, continuous application of conventional pesticides can result in the selection of pesticide-resistant pathogen strains and eventually voids the use of the specific pesticide (Burketova et al., 2015). Synthetic chemical inducers of plant immunity are attractive and promising alternatives. They stimulate or prime the endogenous immunity of plants to combat pathogenic invasions rather than kill the pathogens directly.

Unlike animals that have evolved specific immune cells, nearly each cell in plants is able to act as an “immune cell” to fight against pathogen attacks. Plants can perceive the presence of pathogens through recognition of microbe-associated molecular patterns (MAMPs) or damage-associated molecular patterns (DAMPs) by pattern recognition receptors (PRRs). MAMPs are highly conserved molecular signature within different classes of microbes, for instance, flagellin and elongation factor Tu (EF-Tu) from bacteria, chitin and xylanase from fungi and heptaglucan from oomycetes. DAMPs are plant endogenous immune elicitors released by the pathogen-triggered mechanical stress or enzymatic activities controlled by pathogens, e.g., oligogalacturonides (Schwessinger and Ronald, 2012). The downstream defense activation events following PRR activation include changes of ion fluxes across the plasma membrane, the oxidative burst, activation of mitogen activated protein kinase (MAPK) cascades, gene activation and callose deposition. This MAMPs/DAMPs-triggered immunity (MTI) is the first layer of plant immune system (Jones and Dangl, 2006; Zipfel, 2009). Some pathogens have evolved effectors to interfere with MTI (Dangl et al., 2013). Through co-evolution, plants have developed intracellular immune receptors, Resistance (R) proteins, to recognize the presence of effectors and activate effector-triggered immunity (ETI), which is the second layer of plant immune system (Spoel and Dong, 2012). These two layers of immunity are usually referred to as plant innate immunity (Schwessinger and Ronald, 2012).

The activation of plant innate immunity in local tissue (the infected part) leads to transportation of the mobile defense signals to systemic (uninfected) tissue, resulting in a long-lasting resistance to a broad spectrum of pathogens. This acquired immunity is known as systemic acquired resistance (SAR). The induction of SAR usually confers by ETI, however, it has been reported that MTI can also trigger SAR under some circumstances (Mishina and Zeier, 2007). In addition to pathogens, SAR can be induced by exogenous application of chemical inducers, including salicylic acid (SA), its analogs 2, 6-dichloroisonicotinic acid (INA) and benzothiadiazole S-methyl ester (BTH), its derivatives acetylsalicylic acid (aspirin) and methyl SA (MeSA) (White, 1979; Uknes et al., 1992; Cao et al., 1994; Lawton et al., 1996; Durrant and Dong, 2004; Park et al., 2007), nitric oxide (NO), reactive oxygen species (ROS) (Wang et al., 2014), dicarboxylic acid azelaic acid (AzA) (Jung et al., 2009), the phosphorylated sugar glycerol-3-phosphate (G3P) (Chanda et al., 2011), the abietane diterpenoid dehydroabietinal (DA) (Chaturvedi et al., 2012), the amino-acid derivative pipeolic acid (Pip) (Navarova et al., 2012), and N-hydroxypipecolic acid (NHP) (Chen et al., 2018; Hartmann et al., 2018).

To communicate with the systemic tissue, mobile signals are generated in local tissue and then transported to systemic tissue through phloem. Although, it is well-known that SAR is associated with the accumulation of SA in both local and systemic tissues, grafting studies demonstrated that SA is not the mobile SAR signal (Vernooij et al., 1994). Several chemical candidates for this long-distance signal have been proposed, including MeSA (Park et al., 2007), AzA (Jung et al., 2009), glycerol-3-phosphate (G3P) (Chanda et al., 2011), DA (Chaturvedi et al., 2012), Pip (Navarova et al., 2012), and more recently, its derivative, NHP (Hartmann et al., 2018). Key protein players involved have also been identified including Defective in Induced Resistance 1 (DIR1) (Maldonado et al., 2002; Carella et al., 2017), AzA Insensitive 1 (AZI1) (Jung et al., 2009), and Lipid Transfer Protein 2 (LTP2). Plasmodesmata (PD) is considered to be the transportation route of these signals (Lim et al., 2016). These putative SAR signals might function coordinately to achieve long-distance signal transduction (Dempsey and Klessig, 2012; Shah et al., 2014; Wang et al., 2014).

Once SAR signals are perceived, systemic tissues generate SA to activate a key immune regulator, NON-EXPRESSER OF PR1 (NPR1) to trigger massive transcriptional reprogramming, including the induction of *Pathogenesis-related* (*PR*) genes and endoplasmic reticulum (ER)-resident genes, which aid secretion of PR proteins (Wang et al., 2005, 2006; Spoel and Dong, 2012; Fu and Dong, 2013). Continuous efforts have been made to study the mechanism of how NPR1 responds to SA and regulates downstream defense genes. SA or pathogen infection could cause changes in cellular redox status (Mou et al., 2003). As a result of the cellular redox changes, the cysteine residues of NPR1 (C82 and C216) are reduced by thioredoxins, leading to an oligomer-to-monomer switch in NPR1 conformation and nuclear translocation of the monomer NPR1 (Tada et al., 2008). Nuclear NPR1 monomer then undergoes phosphorylation to promote its transcriptional activity in SAR and its turnover (Spoel et al., 2009). As a transcription co-factor, nuclear NPR1 interacts with TGAs and NIMI-interacting (NIMIN) TFs to regulate the expression of downstream defense genes (Despres, 2003; Kesarwani et al., 2007). TGAs mainly activate NPR1-mediated genes; while NIMIN represses the expression of defense genes (Zhou et al., 2000; Johnson et al., 2003). After fulfillment of its function, ubiquitination of “exhausted” NPR1 leads to its degradation by the proteasomes, allowing “fresh” NPR1 to reinitiate the transcription cycle (Spoel et al., 2009). Recently NPR1 and its paralogs, NPR3 and NPR4, have been found to directly bind SA and serve as its receptors to mediate transcriptional reprogramming (Fu et al., 2012; Wu et al., 2012; Ding et al., 2018). Besides SA, indolic compounds, jasmonic acid (JA), monoterpenes, NO, ROS and intact cuticle also contribute to the establishment of SAR (Truman et al., 2007, 2010; Xia et al., 2009; Navarova et al., 2012; Wendehenne et al., 2014; Riedlmeier et al., 2017).

Induced systemic resistance (ISR) is another form of systemic immunity, which is triggered by non-pathogenic beneficial microbes (Pieterse et al., 2014). Although ISR and SAR are both systemic defense mechanism, they differ in several ways. First, the triggers of ISR and SAR are fundamentally different. SAR is triggered by either compatible or incompatible pathogenic interactions while ISR is initiated by non-pathogenic microbes. Second, although ISR and SAR are both broad-spectrum, their effective spectrum only partially overlaps (Ton et al., 2002). Third, SA is critical to SAR but ISR is less dependent on SA and mainly regulated by JA and ethylene (ET) (Pieterse et al., 1998; Pieterse et al., 2014). Fourth, SAR is accompanied with induction of PR genes and proteins while SA-independent ISR is not (Hoffland et al., 1995). Instead of direct induction of defense machineries, ISR-conditioned plants can elicit faster and/or stronger defenses upon subsequent pathogenic interactions. This sensitization mechanism is called priming (Conrath et al., 2006). It has been shown that priming can reduce the fitness cost associated with constitutive activation of defenses (van Hulten et al., 2006; Walters et al., 2008; Vos et al., 2013). Despite these distinctions between ISR and SAR, SA-independent ISR also depends on NPR1, the key component of SA signaling pathway (Pieterse et al., 1998; Iavicoli et al., 2003; Ryu et al., 2003; Ahn et al., 2007; Hossain et al., 2008; Stein et al., 2008; Segarra et al., 2009; Weller et al., 2012). Cumulating studies suggest that ISR may mainly rely on the cytosolic function of NPR1 while SAR more depends on the nuclear role of NPR1 (Pieterse et al., 2014).

## Synthetic Chemical Inducers of Plant Immunity

Synthetic chemical inducers of plant immunity are structurally different from the natural plant defense elicitors. They may activate or prime plant immunity by simply mimicking the structures of natural immune inducers. Alternatively, they can also be structurally unrelated to natural elicitors and target a subset of defense signaling components. In general, they do not have *in vitro* antimicrobial activity. In this section, we mainly focus on the legacy inducers related to the recently discovered ones, which will be discussed in the “Emerging trends” section.

### SA Derivatives

As a major plant immune hormone, SA plays a pivotal role in the establishment of plant immunity. SA is among the first plant endogenous chemicals reported to induce SAR, which is accompanied by accumulation of PR proteins and resistance to TMV in tomato (White, 1979). In the same study, the famous synthetic SA derivative, Aspirin, was also shown to induce SAR (White, 1979). Later mono- and di-chloro substituted SA derivatives including 4-chloro-SA, 5-chloro-SA and 3, 5-chloro-SA were found to induce PR proteins accumulation and resistance against TMV infection in tobacco (Conrath et al., 1995). More comprehensive investigations of mono- and multiple-substituted SA suggest that 3- and 5-position substitutions are more active than 4- and 6-position substitution. Electron-withdrawing substituents are important to the enhanced activity. Except for 6-fluoro-SA, all fluoro- and chloro-SA tested induced more resistance against TMV than SA (Silverman et al., 2005). Aside from the simple substituted SA, a new class of salicyl glycoconjugates containing hydrazide and hydrazone moieties were synthesized and studied on their *in vitro* and *in vivo* antifungal activity using cucumber (Cui et al., 2014). While the SA hydrazine derivative showed little *in vitro* antifungal activity, significant *in vivo* antifungal activity against *Colletotrichum orbiculare, Fusarium oxysporum, Rhizoctonia solani*, and *Phytophthora capsici* was demonstrated. Intriguingly, while the SA hydrazine derivative is structurally derived from SA, it did not induce the expression of SA marker genes but rather induce JA marker genes. This suggests that the SA hydrazine derivative may not be an SA agonist and function through targeting of other immune signaling components.

### Isonicotinic Acid Derivatives

INA was first identified by Ciba-Geigy, the predecessor of Syngenta, through large-scale screening to identify chemicals that can induce resistance in cucumber against the fungal pathogen *Colletotrichum lagenarium* (Metraux et al., 1991). INA has been shown to induce pathogen resistance in various plants including *Arabidopsis*, tobacco, pear, pepper, rice, cucumber, and beans (Kuc, 1982; Metraux et al., 1991; Ward et al., 1991; Uknes et al., 1992). INA can trigger similar immune responses as SA but independent of SA accumulation as it can still induces SAR in transgenic plants expressing SA hydrolase (*NahG*) in which SA accumulation is compromised (Delaney et al., 1994; Vernooij, 1995). Therefore it functions downstream of the SA accumulation. Recent identifications of SA receptors, NPR3 and NPR4 suggest that INA is likely to be a genuine SA agonist. Similar to SA, INA can also promote the interactions between NPR1 and NPR3. Furthermore, in a competition binding assay, INA was shown to compete with SA to bind its receptors, NPR3 and NPR4 (Fu et al., 2012). Besides the interaction with NPR3 and NPR4, interactions between INA and other SA-binding proteins may also contribute to its role in elicitation of immunity (Durner and Klessig, 1995). However, due to its phytotoxicity effects, INA or its derivatives have not been commercialized for agricultural use.

*N*-cyanomethyl-2-chloro isonicotinic acid (NCI) is another potent plant immune inducer, which belongs to the isonicotinic acid derivative family. It was identified in a screen of 2-chloroisonicotinamide derivatives for control of rice blast (Yoshida et al., 1990a,b). NCI did not show biocidal effects on rice blast *in vitro* even when a high dose was used. However, its *in vivo* antifungal activity against rice blast can last 30 days after a single application. In tobacco, NCI induces expression of PR genes even in *nahG* plants (Nakashita, 2002). This suggests that the immune inducing effect of NCI does not rely on SA accumulation. In *Arabidopsis*, NCI-induced immunity is independent of SA accumulation but depends on NPR1 (Yasuda et al., 2003; Yasuda, 2007). Therefore NCI appears to interact with the signaling steps between SA and NPR1.

### Thiadiazole and Isothiazole Derivatives

BTH is another potent synthetic SAR inducer identified by Ciba-Geigy through a large-scale screening of thiadiazole derivatives (Schurter et al., 1993; Kunz et al., 1997; Oostendorp et al., 2001). BTH does not exhibit antimicrobial activity *in vitro*. However, it can trigger disease resistance against a diverse spectrum of pathogens in various plant species. BTH has been tested in more than 120 pathosystems including resistance in apple and pear against fire blight, tomato against bacterial canker, grapefruit against canker, canola against blackleg disease, cowpea against anthracnose, etc. (Latunde-Dada and Lucas, 2001; Brisset et al., 2002; Soylu et al., 2003; Potlakayala et al., 2007; Graham and Myers, 2011). BTH induces the expression of PR genes and BTH-triggered SAR in *Arabidopsis* is dependent on NPR1 (Lawton et al., 1996). In rice, however, BTH-induced defense responses against rice blast does not require rice ortholog of *Arabidopsis* NPR1 but rather involves WRKY family transcription factor, OsWRKY45 (Shimono et al., 2007). Similar to INA, BTH is also able to induce SAR and expression of PR genes in *nahG* plants (Molina et al., 1998). BTH can be converted by methyl SA esterase to acibenzolar. This conversion is required for BTH-induced PR protein expression as BTH failed to induce PR1 in the methyl SA esterase silenced tobacco seedlings (Tripathi et al., 2010). Besides direct induction of plant defense responses, low doses of BTH can prime plant immunity. In *Arabidopsis*, this priming effect is dependent on NPR1 (Kohler et al., 2002; Goellner and Conrath, 2008). Induction of MAPKs and histone modifications have also been found to associate with and may explain this priming effect (Beckers et al., 2009; Jaskiewicz et al., 2011). Different from INA, BTH has been commercialized as an effective agrochemical.

The isothiazole-based synthetic plant immune inducer, Isotianil, was identified by Bayer AG and Sumitomo Chemical Co., Ltd., through comprehensive search for this type of compounds as protectant against both rice blast and rice blight. Besides rice, Isotianil has also been shown to protect wheat against powdery mildew, cucumber against anthracnose and bacterial leaf spot, Chinese cabbage against Alternaria leaf spot, pumpkin against powdery mildew, strawberry against anthracnose and peach against bacterial shot hole (Ogawa et al., 2011; Krämer et al., 2012). Isotianil does not have antimicrobial activity *in vitro* but relies on its strong immune inducing power to protect rice against rice blast. An exceptionally low dosage is enough to assure its *in vivo* antimicrobial effect (Ogawa et al., 2011). Its effective dose is lower than any other existing plant defense activators (Ogawa et al., 2011). Transcriptome profiling revealed that Isotianil induces the expression of defense-related genes in rice including NPR1, NPR3, and WRKY family transcription factors as well as gene involved in SA catabolism (Krämer et al., 2012). Up till now, more in-depth molecular basis of how Isotianil achieves its immune eliciting activity has not been reported (Maienfisch and Edmunds, 2017).

### JA Analog

While SA regulates defense against biotrophic pathogens, JA and methyl-JA (MeJA) mainly control the immunity against necrotrophic pathogens and herbivores (Santino et al., 2013). JA can be metabolized to MeJA and JA-isoleucine (JA-Ile) which is a biologically active form (Svoboda and Boland, 2010; Pieterse et al., 2012). JA signal is transduced to transcription through JA-Ile triggered degradation of Jasmonate ZIM-domain (JAZ)-type transcriptional repressors by the JA receptor, Coronatine Insensitive 1 (COI1) (Yan et al., 2013, 2018). With the removal of these repressors, JA-responsive genes are de-repressed and JA-dependent defense responses are activated (Browse, 2009; Pieterse et al., 2012; Monte et al., 2014). The phytotoxin, coronatine, is a natural structural and functional mimic of JA-Ile (Weiler et al., 1994; Fonseca et al., 2009). Coronatine can elicit similar responses as JA. In an effort to identify more potent mimics of coronatine, the synthetic JA mimic coronalon was synthesized (Schuler et al., 2001). Coronalon was later shown to mediate stress responses in various plants species (Schuler et al., 2004). It can induce known MeJA-activated defense products as well as MeJA-responsive genes (Pluskota et al., 2007). Besides coronalon, several synthetic JA mimics have been studied and shown to induce JA signaling and defense responses in lima bean, soybean and coyote tobacco (Krumm et al., 1995; Fliegmann et al., 2003; Pluskota et al., 2007). However, whether these JA mimics bind COI1 has not been investigated. Based on the co-receptor structure, a coronatine derivative, coronatine-O-methyloxime (COR-MO), was synthesized through direct chemical derivation and identified as a potent competitive antagonist of jasmonate perception (Monte et al., 2014).

### β-Aminobutyric Acid (BABA)

BABA is a non-protein amino acid that has been known to induce plant resistance since 1963 (Papavizas and Davey, 1963). It has been shown to protect about 40 different plant species against a diverse range of pathogen and pests including virus, bacteria, oomycetes, fungi, nematode, and arthropods (Cohen et al., 2016). BABA primes multiple defense mechanisms regulated by SA-dependent and SA-independent pathways (Zimmerli et al., 2000; Ton et al., 2005). The priming effects elicited by BABA can be maintained to the next generation, making BABA the first plant immune inducer with transgenerational efficacy (Slaughter et al., 2012). BABA is sensed by an aspartyl-tRNA synthetase, IBI1 (Luna et al., 2014). Binding of BABA to IBI1 primes it for alternative defense activity. However, the inhibition of BABA on the aspartyl-tRNA synthetase activity leads to toxicity in plants, which makes BABA unsuitable for agricultural use. While BABA has long been considered as a synthetic plant immune priming agent, a recent study has unequivocally identified BABA as an endogenously metabolite synthesized by various plant species including *Arabidopsis*, Chinese cabbage, maize, teosinte, and wheat (Thevenet et al., 2017).

## Emerging Trends

Large-scale screens performed by the private sector identified the first-generation synthetic elicitors including INA and BTH. Over the last 15 years, advances in combinatorial chemistry and development of high-throughput screening systems have equipped the scientists outside the private sector with the ability to carry out comprehensive screens for synthetic plant immune inducers. This has led to the discovery of a rich arsenal of the second-generation synthetic elicitors (Bektas and Eulgem, 2015). While systematic screens will continue to help us unveil new and better synthetic elicitors, approaches based on the knowledge of known synthetic and/or natural elicitors are emerging.

### Chemical Derivation

Simple chemical derivation of known immune inducers has been and continues to be a shortcut to the identification of more potent immune elicitors. Recently, a new class of SA derivative, benzoylsalicylic acid (BzSA) was identified from seed coats of *Givotia rottleriformis*, a soft-wood tree species (Kamatham et al., 2016). BzSA induces SAR-related gene expression more effectively than SA. It also induced more local and systemic resistance against TMV in tobacco than SA. Through relatively simple chemical derivation, Kamatham et al. (2017) synthesized 14 BzSA derivatives and tested their bioefficacy using the tobacco-TMV pathosystem. When low dosage was tested, all 14 derivatives caused more reduction of the lesion size than both SA and BzSA. The immune-inducing effects of BzSA derivatives are not dependent on SA accumulation as they can still induce resistance in *nahG* plants.

With the availability of a diverse collection of known synthetic and nature plant immune inducers, comparison between known elicitors may help identify specific moiety critical to the immune inducing ability. The 3-methylfuran-containing natural products like menthofuran, furanoeremophilane, caclol, and tanshinone are plant secondary metabolites involved in plant defense (Hägele and Rowell-Rahier, 2000; Maffei et al., 2012; Liu et al., 2013). Based on the prediction that 3-methylfuran moiety may be important to the antimicrobial activity of these secondary metabolites, He et al. (2017) used diversity-oriented synthesis to generate a small natural-products-like library containing the 3-methylfuran scaffold. Five 3-methylfuran derivatives were found to significantly induce the resistance in rice against brown planthopper, supporting the initial speculation on the critical role of 3-methylfuran (He et al., 2017).

Besides specific functional moiety, the pattern of known immune elicitors can also be useful information for the design of new ones. Rhamnolipids and lipopeptides have been found as a new class of MAMPs (Jourdan et al., 2009; Sanchez et al., 2012; Farace et al., 2015). Both rhamnolipids and lipopeptides are amphiphilic compounds. Due to the biocompatibility and biodegradability, rhamnoside-based bolaamphiphiles surfactants have been increasingly recognized and investigated (Gatard et al., 2013; Akong and Sandrine, 2015). The bolaamphiphiles surfactants contains a long hydrophobic spacer connecting two hydrophilic moieties. Luzuriaga-Loaiza et al. (2018) synthesized rhamnolipid bolaforms (SRBs) and tested their immune induction activity. Depending on the acyl chain length, SRBs differentially induce defense responses and confer local resistance in *Arabidopsis* against the hemibiotrophic bacteria *Pseudomonas* *syringae* but not the necrotrophic fungal pathogen *Botrytis cinerea*.

Chemical derivation based on known natural immune inducers has great expedited the invention of better synthetic immune inducers. However, the lack of the mechanistic understanding of the interactions between the new synthetic immune inducers and their cognate targets in plants has limited our ability to improve the efficacy or lower the phytotoxicity in a more rational manner. More comprehensive biochemical studies using the new synthetic immune inducers will provide a promising guide.

### Bifunctional Combination

Bifunctional combination approaches combine a known synthetic plant immune inducer with another compound, which brings other functions to the final product. Strobilurins are a class of broad spectrum fungicides. Widespread use of strobilurins have caused pathogen resistance (Gisi et al., 2002; Leiminger et al., 2014). 3,4-dichloroisothiazole derivatives have diverse biological activities including immune-inducing activity. For example, as mentioned in Section 2.3, Isotianil, a 3,4-dichloroisothiazole derivative, is a very potent immune elicitor. In an effort to identify new strobilurins for future market, Chen et al. (2017) combined 3,4-dichloroisothiazoles with strobilurins. Through the incorporation of 3,4-dichloroisothiazole, new strobilurins with good in *vivo* and *in vitro* fungicide activities were identified.

JA-Ile is a natural conjugation of JA and isoleucine and was previously identified as the sole endogenous bioactive JA molecule. In an effort to identify additional endogenous bioactive jasmonates, Yan et al. (2016) coupled 20 natural amino acids with coronafacic acid (CFA) which is a part of the phytotoxic natural JA-Ile mimic, coronatine, and identified 5 non-polar amino acid conjugates of CFA including CFA-Ile, CFA-Leu, CFA-Val, CFA-Met, and CFA-Ala as new synthetic JA signaling pathway elicitors. Following these findings, JA-Leu, JA-Val, JA-Met, and JA-Ala were further discovered as new endogenous bioactive JA molecules. Through integration of the structural information of all these bioactive JA molecules, general rules of bioactive JA conjugates were proposed. Based on these rules, two additional JA signaling pathway elicitors, CFA-N-Leu and CFA-Ch-Gly were identified (Yan et al., 2016).

Besides covalent combination, ionic pairing is another attractive method, since one can choose ions independently. The same plant immune inducer can be paired with surfactant-type cation for better wetting or tetrabutylammonium cation for faster dissolution. Using this strategy, 15 immunity inducers including SA, BTH, INA, BABA, etc., were paired with the cholinium cation to form ionic liquids (Kukawka et al., 2018). Their abilities to induce SAR were tested using the tobacco-TMV pathosystem. Cholinium is an essential nutrient. Pairing with cholinium reduced phytotoxicity of these immune inducers while only mild perturbation to the immune-inducing ability was recorded.

While bifunctional combination approaches have shown the potential to either improve the efficacy or reduce phytotoxicity, the introduction of the second chemical moiety has also brought more complications. For example, Pip, a SAR mobile signal candidate, showed significantly reduced SAR-inducing activity when paired with cholinium (Kukawka et al., 2018). On the other hand, while isonicotinate did not induce SAR, its cholinium ionic liquid was shown to induce SAR (Kukawka et al., 2018). Therefore bifunctional combination is not merely the addition of the biological activities of the two chemical moieties but rather results in potentially complicated interactions between the signaling pathways induced by the two moieties. Careful characterization is thus essential to understand the full spectrum of the biological activities of the new synthetic immune inducers identified through bifunctional combination approaches.

### Computer-Aided Design

Manual inspection can only process a handful of immune elicitors for recognition of potentially critical bioactive substructures and patterns of known immune inducers (He et al., 2017; Luzuriaga-Loaiza et al., 2018). Advances in high-performance computing have made it possible to screen tens of thousands of lead-like molecules computationally. This computer-aided design (CAD) drug design strategy has been increasingly recognized and utilized in pesticide discovery and property analysis (Xia et al., 2014; Veselinovi et al., 2015; Burden et al., 2016). Using SA, MeSA, BTH, and Tiadinil, the four known immune inducers as query templates, Chang et al. (2017) performed virtual screening against the 5,3000 hit-like and lead-like compounds in the Maybridge database and identified three benzotriazole scaffolds as promising leading compounds. One of them, L1 shows high 3D structure similarity to BTH despite their differences in 2D topology. Furthermore, L1 also shares similar pharmacophore features to BTH. *In vivo* screening of L1 derivatives identified new immune inducers with comparable or improved efficacy against *Mycosphaerella* *melonis, Corynespora cassiicola, P. syringae, B. cinerea*, and *F. oxysporum* in cucumber, *Phytophthora infestans* in tomato and *R. solani* in rice.

Besides the knowledge of the small lead-like compounds, structural understanding of plant receptors can also lend power to the virtual screening of new leading compounds. Using the high quality structural model of JA receptor, COI1, 767 JA analogs were analyzed in terms of their ability to bind COI1 (Pathak et al., 2017). Two such analogs ZINC27640214 and ZINC43772052 showed higher binding affinity compared to JA. ZINC27640214 appears to have efficient, stable and good cell permeability properties, making it a good candidate for experimental validation. Buswell et al. (2018) combined the knowledge of the structural information on the BABA receptor, IBI1 and small-scale screening of β-amino acids using the *ibi1* mutant to search for BABA analogs, which induce plant immunity without severe growth inhibition. Out of the seven resistance-inducing compounds, five of them showed no inhibition on growth. Among these five, (R)-β-homoserine (RBH) showed the strongest resistance-inducing activity without affecting vegetative growth or global plant metabolism. Interestingly, RBH appears to elicit partially different signaling pathways from those affected by BABA, making it a promising new crop protectant. Through *in silico* docking and subsequent molecular dynamics simulation, the keto group of a stereoisomer of coronatine showed the potential to control the binding selectivity between its derivatives and different subtypes of JAZ (Takaoka et al., 2018). An oxime derivative of this coronatine stereoisomer was then developed as a synthetic JAZ subtype-selective agonist, specifically targeting JAZ9 and JAZ10. This selectivity in JAZ enabled induction of pathogen resistance without a cost on growth. It is noteworthy that small-scale targeted characterization of synthetic agonist candidates rather than large-scale screening was realized in this study owing to the integration of the structural information on both the ligands and the receptor.

As an emerging trend, application of CAD in the discoveries of new synthetic immune inducers awaits further exploitation. While lead-like compound databases have provided a critical foundation for virtual chemical screening, they also restrain the chemical diversity and may potentially hinder the discovery of completely novel scaffolds. On the other side, CAD based on the structural information of plant defense signaling components does not set a limit on the chemical diversity. However, synthetic immune activators identified through this route may be only effective in the specific plant species studied due to the sequence variation among different plant species. Integration of evolutional conservation information may help alleviate this issue.

## Conclusion and Perspectives

In this review, we provided a focused overview on the discovery and functional properties of synthetic plant immune inducers and emerging trends in the search for new and improved synthetic inducers. A rich knowledge of the structural, chemical and pharmacological properties of the known inducers has opened up some shortcuts to expedite the discovery procedure. Instead of *in vivo* screening tens of thousands small molecules, small-scale screening involves only a few dozens or even a handful of compounds is able to identify new inducer derivatives or even completely new scaffolds through integration of prior knowledge. While the availability of the structures of small compounds is the major drive for this advancement, we anticipate that integration of more prior information will greatly facilitate the discovery of novel and better plant immune elicitors. This includes the structural information, biological function and evolutional conservation of key plant immune-related signaling components, physical and biochemical features of the small compounds as well as the structural basis and evolutional conservation of the molecular interactions between small compounds and their cognate plant immune signaling components.

The great expansion of synthetic immune inducers has also provided opportunities to dissect the signaling networks of plant immune system that is not accessible to genetic screens due to the lethality and gene redundancy. With the discovery of the hidden drug-able targets in plant immune system, new synthetic immune inducers may be developed to target these hidden points. Then in turn, these new inducers can again enhance our ability to dissect plant immune system and keep this discovery cycle going on.

## Author Contributions

All authors listed have made a substantial, direct and intellectual contribution to the work, and approved it for publication.

## Conflict of Interest Statement

The authors declare that the research was conducted in the absence of any commercial or financial relationships that could be construed as a potential conflict of interest.
